# Supplementary material for: Resolving the full spectrum of human genome variation using Linked-Reads
Source: Genome Res. 2019 Apr;29(4):635–45. doi: 10.1101/gr.234443.118 (PMC6442396; doi:10.1101/gr.234443.118)
Supplement: Supplemental Material [file supp_gr.234443.118_Supplemental_longranger-2.2.zip › longranger-2.2.tar.bz2!/lr2.2-public/lib/stan/cmdstan/stan/doc/api/html/ast_8hpp.html]

Stan: src/stan/gm/ast.hpp File Reference


|  |  |
| --- | --- |
|  | Stan  2.5.0  probability, sampling & optimization |


- Main Page
- Related Pages
- Namespaces
- Classes
- Files

- File List
- File Members

All Classes Namespaces Files Functions Variables Typedefs Enumerations Enumerator Friends Macros Pages

- src
- stan
- gm

Classes |
Namespaces |
Typedefs |
Functions |
Variables

ast.hpp File Reference

`#include <map>`  
`#include <string>`  
`#include <vector>`  
`#include <set>`  
`#include <boost/variant/recursive_variant.hpp>`

Go to the source code of this file.

|  |  |
| --- | --- |
| Classes | |
| struct | stan::gm::nil |
|  | Placeholder struct for boost::variant default ctors. More... |
|  | |
| struct | stan::gm::expr\_type |
|  | |
| class | stan::gm::function\_signatures |
|  | |
| struct | stan::gm::statements |
|  | |
| struct | stan::gm::distribution |
|  | |
| struct | stan::gm::expression\_type\_vis |
|  | |
| struct | stan::gm::expression |
|  | |
| struct | stan::gm::printable |
|  | |
| struct | stan::gm::is\_nil\_op |
|  | |
| struct | stan::gm::variable\_dims |
|  | |
| struct | stan::gm::int\_literal |
|  | |
| struct | stan::gm::double\_literal |
|  | |
| struct | stan::gm::array\_literal |
|  | |
| struct | stan::gm::variable |
|  | |
| struct | stan::gm::integrate\_ode |
|  | |
| struct | stan::gm::fun |
|  | |
| struct | stan::gm::index\_op |
|  | |
| struct | stan::gm::binary\_op |
|  | |
| struct | stan::gm::unary\_op |
|  | |
| struct | stan::gm::range |
|  | |
| struct | stan::gm::base\_var\_decl |
|  | |
| struct | stan::gm::variable\_map |
|  | |
| struct | stan::gm::int\_var\_decl |
|  | |
| struct | stan::gm::double\_var\_decl |
|  | |
| struct | stan::gm::unit\_vector\_var\_decl |
|  | |
| struct | stan::gm::simplex\_var\_decl |
|  | |
| struct | stan::gm::ordered\_var\_decl |
|  | |
| struct | stan::gm::positive\_ordered\_var\_decl |
|  | |
| struct | stan::gm::vector\_var\_decl |
|  | |
| struct | stan::gm::row\_vector\_var\_decl |
|  | |
| struct | stan::gm::matrix\_var\_decl |
|  | |
| struct | stan::gm::cholesky\_factor\_var\_decl |
|  | |
| struct | stan::gm::cholesky\_corr\_var\_decl |
|  | |
| struct | stan::gm::cov\_matrix\_var\_decl |
|  | |
| struct | stan::gm::corr\_matrix\_var\_decl |
|  | |
| struct | stan::gm::name\_vis |
|  | |
| struct | stan::gm::var\_decl |
|  | |
| struct | stan::gm::statement |
|  | |
| struct | stan::gm::is\_no\_op\_statement\_vis |
|  | |
| struct | stan::gm::returns\_type\_vis |
|  | |
| struct | stan::gm::increment\_log\_prob\_statement |
|  | |
| struct | stan::gm::for\_statement |
|  | |
| struct | stan::gm::conditional\_statement |
|  | |
| struct | stan::gm::while\_statement |
|  | |
| struct | stan::gm::print\_statement |
|  | |
| struct | stan::gm::reject\_statement |
|  | |
| struct | stan::gm::return\_statement |
|  | |
| struct | stan::gm::no\_op\_statement |
|  | |
| struct | stan::gm::arg\_decl |
|  | |
| struct | stan::gm::function\_decl\_def |
|  | |
| struct | stan::gm::function\_decl\_defs |
|  | |
| struct | stan::gm::program |
|  | |
| struct | stan::gm::sample |
|  | |
| struct | stan::gm::assignment |
|  | |
| struct | stan::gm::contains\_var |
|  | |
| struct | stan::gm::contains\_nonparam\_var |
|  | |

|  |  |
| --- | --- |
| Namespaces | |
|  | stan |
|  | Probability, optimization and sampling library. |
|  | |
|  | stan::gm |
|  | Directed graphical model compiler. |
|  | |

|  |  |
| --- | --- |
| Typedefs | |
| typedef int | stan::gm::base\_expr\_type |
|  | |
| typedef std::pair< expr\_type,   std::vector< expr\_type > > | stan::gm::function\_signature\_t |
|  | |
| typedef int | stan::gm::var\_origin |
|  | |

|  |  |
| --- | --- |
| Functions | |
| std::ostream & | stan::gm::write\_base\_expr\_type (std::ostream &o, base\_expr\_type type) |
|  | |
| std::ostream & | stan::gm::operator<< (std::ostream &o, const expr\_type &et) |
|  | |
| expr\_type | stan::gm::promote\_primitive (const expr\_type &et) |
|  | |
| expr\_type | stan::gm::promote\_primitive (const expr\_type &et1, const expr\_type &et2) |
|  | |
| bool | stan::gm::is\_nil (const expression &e) |
|  | |
| size\_t | stan::gm::total\_dims (const std::vector< std::vector< expression > > &dimss) |
|  | |
| expr\_type | stan::gm::infer\_type\_indexing (const base\_expr\_type &expr\_base\_type, size\_t num\_expr\_dims, size\_t num\_index\_dims) |
|  | |
| expr\_type | stan::gm::infer\_type\_indexing (const expression &expr, size\_t num\_index\_dims) |
|  | |
| void | stan::gm::print\_var\_origin (std::ostream &o, const var\_origin &vo) |
|  | |
| bool | stan::gm::returns\_type (const expr\_type &return\_type, const statement &statement, std::ostream &error\_msgs) |
|  | |
| void | stan::gm::generate\_expression (const expression &e, std::ostream &o) |
|  | |
| bool | stan::gm::has\_rng\_suffix (const std::string &s) |
|  | |
| bool | stan::gm::has\_lp\_suffix (const std::string &s) |
|  | |
| bool | stan::gm::is\_user\_defined (const std::string &name, const std::vector< expression > &args) |
|  | |
| bool | stan::gm::is\_user\_defined\_prob\_function (const std::string &name, const expression &variate, const std::vector< expression > &params) |
|  | |
| bool | stan::gm::is\_user\_defined (const fun &fx) |
|  | |
| bool | stan::gm::has\_var (const expression &e, const variable\_map &var\_map) |
|  | |
| bool | stan::gm::has\_non\_param\_var (const expression &e, const variable\_map &var\_map) |
|  | |
| bool | stan::gm::is\_assignable (const expr\_type &l\_type, const expr\_type &r\_type, const std::string &failure\_message, std::ostream &error\_msgs) |
|  | |
| bool | stan::gm::ends\_with (const std::string &suffix, const std::string &s) |
|  | |

|  |  |
| --- | --- |
| Variables | |
| const int | stan::gm::VOID\_T = 0 |
|  | |
| const int | stan::gm::INT\_T = 1 |
|  | |
| const int | stan::gm::DOUBLE\_T = 2 |
|  | |
| const int | stan::gm::VECTOR\_T = 3 |
|  | |
| const int | stan::gm::ROW\_VECTOR\_T = 4 |
|  | |
| const int | stan::gm::MATRIX\_T = 5 |
|  | |
| const int | stan::gm::ILL\_FORMED\_T = 6 |
|  | |
| const int | stan::gm::model\_name\_origin = 0 |
|  | |
| const int | stan::gm::data\_origin = 1 |
|  | |
| const int | stan::gm::transformed\_data\_origin = 2 |
|  | |
| const int | stan::gm::parameter\_origin = 3 |
|  | |
| const int | stan::gm::transformed\_parameter\_origin = 4 |
|  | |
| const int | stan::gm::derived\_origin = 5 |
|  | |
| const int | stan::gm::local\_origin = 6 |
|  | |
| const int | stan::gm::function\_argument\_origin = 7 |
|  | |
| const int | stan::gm::function\_argument\_origin\_lp = 8 |
|  | |
| const int | stan::gm::function\_argument\_origin\_rng = 9 |
|  | |
| const int | stan::gm::void\_function\_argument\_origin = 10 |
|  | |
| const int | stan::gm::void\_function\_argument\_origin\_lp = 11 |
|  | |
| const int | stan::gm::void\_function\_argument\_origin\_rng = 12 |
|  | |


---

[ Stan Home Page ]

*© 2011–2014,
Stan Development Team.*
